# Chemical Composition and Biological Activity of Essential Oils of *Origanum vulgare* L. subsp. *vulgare* L. under Different Growth Conditions

**DOI:** 10.3390/molecules181214948

**Published:** 2013-12-04

**Authors:** Enrica De Falco, Emilia Mancini, Graziana Roscigno, Enrico Mignola, Orazio Taglialatela-Scafati, Felice Senatore

**Affiliations:** 1 Department of Pharmacy, University of Salerno, Via Giovanni Paolo II, 84084 Fisciano (SA), Italy; E-Mails: edefalco@unisa.it (E.D.F.); emancini@unisa.it (E.M.); groscigno@unisa.it (G.R.); 2 Department of Pharmacy, University of Naples “Federico II”, Via D. Montesano 49, 80131 Napoli, Italy; E-Mails: enri.mignola@gmail.com (E.M.); felice.senatore@unina.it (F.S.)

**Keywords:** *Origanum vulgare* subsp. *vulgare*, plant distribution, essential oil composition, antimicrobial activity

## Abstract

This research was aimed at investigating the essential oil production, chemical composition and biological activity of a crop of pink flowered oregano (*Origanum vulgare* L. subsp. *vulgare* L.) under different spatial distribution of the plants (single and binate rows). This plant factor was shown to affect its growth, soil covering, fresh biomass, essential oil amount and composition. In particular, the essential oil percentage was higher for the binate row treatment at the full bloom. The chemical composition of the oils obtained by hydrodistillation was fully characterized by GC and GC-MS. The oil from plants grown in single rows was rich in sabinene, while plants grown in double rows were richer in ocimenes. The essential oils showed antimicrobial action, mainly against Gram-positive pathogens and particularly *Bacillus cereus* and *B. subtilis*.

## 1. Introduction

Aromatic plants are considered of great interest for their flavours and for their medicinal properties, along with human consumption, animal foodstuff and ornamental uses; thus, they are especially suitable for multifunctional sustainable crop models [1,2,3,4]. A large number of these aromatic species belong to the family Lamiaceae, whose centre of differentiation is located in the Mediterranean area. Within this family, oregano (*Origanum vulgare* subsp. *vulgare*) is probably one of most widely used aromatic plant, whose essential oils are particularly rich in mono- and sesquiterpenes [5].

Oregano essential oils have been shown to possess antioxidant, antibacterial, antifungal, diaphoretic, carminative, antispasmodic and analgesic activities [6,7,8,9,10,11] and, among these, the antimicrobial potential is of special interest. In recent years, a large number of researches have reported the efficacy of essential oils from several *Origanum* species against a panel of bacterial strains [12,13,14,15], and Başer *et al.* identified carvacrol as the main responsible for this biological activity [16].

In this context, and in view of a possible use of a possible use of oregano oils as antimicrobial herbal medicines, it is important to consider that the essential oil yield and composition are the result of different factors [5], including genotype [17], environment [18], developmental stage [19] and cultural practices [20,21,22].

Interestingly, Halva [23] and Piccaglia [24] have analyzed *Anethum* and *Lavandula* species, respectively, and reported that essential oil yields and composition are affected by spatial plant distribution and density of plantation, factors that are supposed to have impact on plant growth and light interception. Conversely, some research conducted in Sicily (Italy) compared different plant densities for *O. vulgare* L. subsp. *hirtum* (Link) Ietswaart, and did not indicate significant differences in the yield and composition of the essential oils [25].

The aim of the present research was to investigate growth, essential oil production and chemical composition of a crop of pink flowered oregano (*Origanum vulgare* L. subsp. *vulgare* L.) under different plant spatial distribution scenarios (binate and single rows). Moreover, the antimicrobial activity of the essential oil in relation to the chemical composition was investigated.

## 2. Results and Discussion

### 2.1. Results of Field Experiment

This research started at the beginning of 2007 on plants of *Origanum vulgare* L. subsp. *vulgare* L. (sin. *O. vulgare* L.) coming from a commercial nursery after three years from the transplantation on a deep clay sandy soil. The thermo-precipitation trends during the period of the experiment in 2007 showed minimum temperatures always above 0 °C. As for the maximum temperatures, the values reached 30 °C during the last ten days of June and maintained this pattern until the last week of August. Rainfall totaled 212 mm during the winter period, 88 mm during the spring and was absent during the months of July and August. This thermo-precipitation tendency implied that watering was only required in the second decade of May.

The growth parameters measured during the field experiments are reported in Table 1. As expected, the growth of oregano was influenced by the spatial distribution of the plants. The height of vegetation was higher in the binate row until the beginning of the bloom, probably due to the competition among the plants in the bine. The data about the width of the vegetation showed that the plants allowed very early the full covering of the soil in the single rows distanced 40 cm. On the contrary, in the other treatment, the vegetation didn’t cover completely the soil also at the stage of maximum growth due to the high distance between the bine (120 cm). Anyway, the PAR absorbance on the row reached the maximum value at the beginning of the bloom in both treatments and this showed a high efficiency of the vegetation in the interception of the radiation. The values of the fresh biomass per plant were higher for the binate treatment and this can be explained on the basis of the greater available space for every plant. On the contrary, the values of fresh biomass per m^2^ were always higher in the single row treatment. Thus, we can assume that the greatest fresh biomass per plant in the binate row treatment was not sufficient to recover from the lower number of plants per m^2^.

**Table 1:** Results related to the parameters measured during the growth of oregano in the field experiment.

Measured parameters	Rows	Biological phases					
		Growth (April 19th)		Bloom beg. (May 24th)		Full bloom (June 18th)	
Vegetation height (cm)	single	14.2	b	53.8	b	84.2	a
	binate	18.3	a	62.4	a	75.6	a
Row width	single	44.8	b	46.2	b	45.6	b
(cm)	binate	70.7	a	109.5	a	106.6	a
PAR absorbance	single	26	b	100	a	96	b
(%)	binate	18	a	100	a	98	a
Fresh biomass	single	192	b	422	b	569	b
(g per plant)	binate	289	a	662	a	696	a
Fresh biomass	single	963	a	2111	a	2846	a
(g m^−2^)	binate	830	b	1921	b	2020	b
Essential oil	single	0.007	a	0.018	a	0.030^a^	b
(% fresh biomass)	binate	0.009	a	0.012	a	0.051^a^	a

^a^ = sample of essential oil submitted to chemical analysis. Equal letters pointed out equal values per *p* ≥ 0.05 at LSD test.

The essential oil/fresh biomass ratios were in full agreement with those already reported for these species and increased during the biological cycle, reaching the maximum values at the full bloom [26,27]. The values were higher for the binate row treatment only at the full bloom and this can be explained by the high ability of the vegetation to intercept the PAR in both treatments, as previously pointed out. Moreover, we can suppose that the conditions of the growth which led to the greater biomass per plant in the binate treatment contributed to increase the essential oil percentage, given the significant correlation between the biomass/plant ratio and the essential oil percentage (0.7*). Similar behavior was detected for the oil percentage obtained from the air dried samples; in fact, the value was higher for the binate treatment compared to the single row (0.18% and 0.13% respectively; LSD at *p* ≥ 5% = 0.003).

**Table 2:** Chemical composition of the essential oils from *Origanum vulgare* L. subsp. *vulgare* L. samples at full bloom.

Essential oil samples		Plant disposal		Single row		Binate row	
		Oregano biomass		Fresh	Dried	Fresh	Dried
RI ^a^	RI ^b^	Component	Id. ^c^	1% ^d^	% 2 ^d^	% 3 ^d^	% 4 ^d^
		**Monoterpene hydrocarbons**		**18.1**	**14.0**	**19.4**	**16.7**
931	1023	α-Thujene	1, 2	0.2	0.2	t	0.3
938	1032	α-Pinene	1, 2, 3	0.5	0.3	0.1	0.4
973	1132	Sabinene	1, 2	9.1	4.8	2.5	3.2
993	1174	Myrcene	1, 2, 3	0.5	0.7	1.2	0.6
1001	1146	δ^2^-Carene	1, 2	0.3	0.5	0.2	1.1
1005	1150	α-Phellandrene	1, 2, 3				0.5
1012	1189	α-Terpinene	1, 2, 3				t
1020	1187	*o-*Cymene	1, 2		0.2		
1025	1278	*p*-Cymene	1, 2, 3	3.9	3.8	3.3	4.1
1029	1218	β-Phellandrene	1, 2				0.7
1030	1203	Limonene	1, 2, 3	0.8			
1038	1245	(*Z*)-β-Ocimene	1, 2	0.8	0.8	4.0	1.9
1049	1262	(*E*)-β-Ocimene	1, 2	0.9	0.8	3.1	1.5
1057	1256	γ-Terpinene	1, 2, 3	0.3	0.6	0.9	1.5
1086	1265	Terpinolene	1, 2, 3	0.1	0.2	0.1	0.4
1129	1386	*allo*-Ocimene	1, 2	1.1	1.1	4.0	0.6
		**Oxygenated monoterpenes**		**7.2**	**15.5**	**3.9**	**17.6**
1034	1213	1,8-Cineole	1, 2, 3	0.6	3.9		2.6
1059	1555	(*Z*)-Sabinyl acetate	1, 2	0.4	0.2	0.1	0.6
1076	1450	(*Z*)-Linalool oxide (furanoid)	1, 2		0.1	t	0.3
1093	1474	*trans*-Sabinene hydrate	1, 2	0.1	0.2	t	0.7
1098	1553	Linalool	1, 2, 3	0.7	0.9	0.8	2.0
1117	1571	*trans*-*p*-Menth-2-en-1-ol	1, 2	0.2	0.3	0.1	0.6
1128	1638	*cis*-*p*-Menth-2-en-1-ol	1, 2	t	0.4		0.6
1141	1684	*trans*-Verbenol	1, 2		0.1		
1145	1532	Camphor	1, 2, 3				t
1155	1652	Sabina ketone	1, 2		0.3		0.5
1167	1719	Borneol	1, 2, 3		0.2	0.1	0.4
1175	1583	*cis*-Isopulegone	1, 2	0.4	t	0.3	0.2
1176	1611	Terpinen-4-ol	1, 2, 3	3.5	5.6	1.9	5.0
1183	1758	*cis*-Piperitol	1, 2	0.1	0.1		
1189	1706	α-Terpineol	1, 2, 3	0.9	2.3	0.6	2.3
1193	1648	Myrtenal	1, 2				0.1
1196	1804	Myrtenol	1, 2		0.2		0.3
1207	1689	*trans*-Piperitol	1, 2	0.1	0.1		0.2
1218	1802	Cuminaldehyde	1, 2		0.2		
1238	1662	Pulegone	1, 2		0.1		0.8
1242	1752	Carvone	1, 2	0.2		t	
1265	1680	*cis*-Piperitone oxide	1, 2				0.1
1275	1744	Phellandral	1, 2		0.1		0.1
1288	2113	Cumin alcohol	1, 2		0.1		0.2
1315	2073	*p*-Mentha-1,4-dien-7-ol	1, 2		0.1		
		**Sesquiterpene hydrocarbons**		**34.2**	**22.9**	**43.9**	**16.5**
1339	1494	Bicycloelemene	1, 2	0.2	0.5	1.8	0.5
1348	1466	α-Cubebene	1, 2		0.1	0.1	0.2
1377	1497	α-Copaene	1, 2	0.2	0.2	0.2	0.9
1382	1547	β-Cubebene	1, 2				0.2
1385	1535	β-Bourbonene	1, 2	0.7	1.5	1.1	1.0
1387	1600	β-Elemene	1, 2	0.6	0.3	0,8	0.2
1408	1529	α-Gurjunene	1, 2			0.1	0.1
1415	1612	β-Caryophyllene	1, 2, 3	15.6	8.8	17.2	5.3
1432	1612	β-Gurjunene	1, 2	0.3			
1432	1650	γ-Elemene	1, 2		1.3		
1398	1685	α-Elemene	1, 2				t
1437	1628	Aromadendrene	1, 2	0.1		0.1	
1438	1573	*trans*-α-Bergamotene	1, 2			1.7	0.3
1452	1673	(*E*)-β-Farnesene	1, 2	t			
1455	1689	α-Humulene	1, 2	2.1	1.1	2.8	0.8
1463	1667	*allo*-Aromadendrene	1, 2	0.5	0.8	0.8	0.7
1477	1726	Germacrene D	1, 2	4.5	3.3	9.8	2.1
1478	1679	α-Amorphene	1, 2		t		
1478	1704	γ-Muurolene	1, 2	0.4	0.4	0.8	
1483	1784	*ar*-Curcumene	1, 2	t			
1489	1734	*epi*-Bicyclosesquiphellandrene	1, 2	1.0	0.8	1.9	0.7
1490	1694	*cis* β-Guajene	1, 2	0.1	0.1	0.2	0.2
1492	1756	Bicyclogermacrene	1, 2	0.8		2.9	0.1
1494	1740	Valencene	1, 2				t
1503	1740	α-Muurolene	1, 2	0.6	0.3	0.7	0.7
1506	1758	(*E,E*)-α-Farnesene	1, 2	2.6	0.3		0.6
1510	1743	β-Bisabolene	1, 2		0.7		0.2
1515	1776	γ-Cadinene	1, 2	1.4		1.3	0.5
1519	1839	1-S-*cis*-Calamenene	1, 2			t	
1526	1773	δ-Cadinene	1, 2	2.4	1.7	0.2	1.0
1532	1745	α-Cadinene	1, 2	0.2	0.2	0.3	0.1
1541	1918	α-Calacorene	1, 2			0.1	0.1
1544	1854	Germacrene B	1, 2		0.7		
		**Oxygenated sesquiterpenes**		**16.7**	**24.9**	**20.6**	**20.0**
1520	2048	*endo*-1-Bourbonanol	1, 2	0.2			0.3
1553	2076	*cis*-α-Copaen-8-ol	1, 2		0.4		
1565	2057	Ledol	1, 2	0.3	0.4	0.2	0.6
1575	2069	Germacrene D 4-ol	1, 2	t		t	
1577	2150	Spathulenol	1, 2, 3	6.1	18.6	5.3	9.4
1579	2008	Caryophyllene oxide	1, 2, 3	0.2	1.4	1.1	2.9
1587	2098	Globulol	1, 2	0.8		1.0	1.0
1591	2104	Viridiflorol	1, 2	0.8	0.7	1.0	0.6
1621	2324	Caryophylladienol II	1, 2				0.4
1636	2183	γ-Eudesmol	1, 2	5.0		6.7	1.0
1638	2316	Caryophylladienol I	1, 2		0.6		
1640	2158	t-Cadinol	1, 2	1.0	0.7	2.1	0.8
1642	2209	t-Muurolol	1, 2	1.6		3.2	0.6
1645	2208	Torreyol	1, 2	0.7	0.4		
1650		*allo*-Aromadendrene oxide I	1, 2				0.4
1652	2255	α-Cadinol	1, 2		1.7		2.0
		**Phenols**		**1.4**	**12.5**	**0.2**	**16.6**
1239	1609	Thymol methyl ether	1, 2, 3			t	0.3
1245	1975	Carvacrol methyl ether	1, 2, 3		0.2		0.9
1293	2198	Thymol	1, 2, 3		0.5	0.1	1.1
1299	2239	Carvacrol	1, 2, 3	1.4	11.7	0.1	14.3
1353	2186	Eugenol	1, 2, 3		0.1		
		**Carbonylic compounds**		**0.1**	**0.4**	**0.5**	**0.6**
975	1312	1-Octen-3-one	1, 2	0.1		0.5	0.3
1085	1690	Cryptone	1, 2		t		0.1
1395	1969	*cis*-Jasmone	1, 2		0.1		
1482	1958	(*E*)-β-Ionone	1, 2, 3		0.2		
1835	2131	Hexahydrofarnesyl acetone	1, 2		0.1	t	0.2
		**Hydrocarbons**		**14.5**	**1.2**	**0.8**	**3.5**
800	800	Octane	1, 2, 3		t		
2100	2100	Heneicosane	1, 2, 3	0.2			
2200	2200	Docosane	1, 2, 3	0.1		t	
2300	2300	Tricosane	1, 2, 3	0.4			0.2
2400	2400	Tetracosane	1, 2, 3	0.4	0.1	t	0.2
2500	2500	Pentacosane	1, 2, 3	0.8	0.2	0.1	0.4
2600	2600	Hexacosane	1, 2, 3	1.1	0.1	t	0.2
2700	2700	Heptacosane	1, 2, 3	1.5	0.2	0.1	0.5
2800	2800	Octacosane	1, 2, 3	1.7	0.1	0.1	0.4
2900	2900	Nonacosane	1, 2, 3	2.5	0.2	0.2	0.5
3000	3000	Triacontane	1, 2, 3	2.2	t	0.1	0.4
3100	3100	Entriacontane	1, 2, 3	1.7	0.2	0.2	0.4
3200	3200	Dotriacontane	1, 2, 3	1.1	t	t	0.2
3300		Tritriacontane	1, 2	0.5	0.1	t	0.1
3400		Tetratriacontane	1, 2	0.2		t	
3500		Pentatriacontane	1, 2	0.1		t	
		**Others**		**1.0**	**2.5**	**1.1**	**1.7**
980	1454	1-Octen-3-ol	1, 2, 3	0.4	1.3	0.4	1.2
992	1394	Octan-3-ol	1, 2, 3	0.1	0.3		
1286	1485	Dihydroedulan II	1, 2	0.3	0.9	0.4	0.5
1950	2622	(*Z*)-Phytol	1, 2	0.2		0.3	T
1957	2931	Hexadecanoic acid	1, 2, 3			t	
		**TOTAL**		**93.2**	**93.9**	**90.4**	**93.2**

^a^ retention indices relative to C_8_-C_32_ *n*-alkanes on the HP 5MS column; ^b^ retention indices relative to C_8_-C_32_ *n*-alkanes on the HP Innowax column; ^c^ Identification: 1: retention index identical to literature, 2: comparison of mass spectra with MS libraries, 3: co-injection with authentic compounds; ^d^ t: trace, concentration less than 0.05%.

### 2.2. Chemical Composition of the Essential Oil

**Figure 1:**
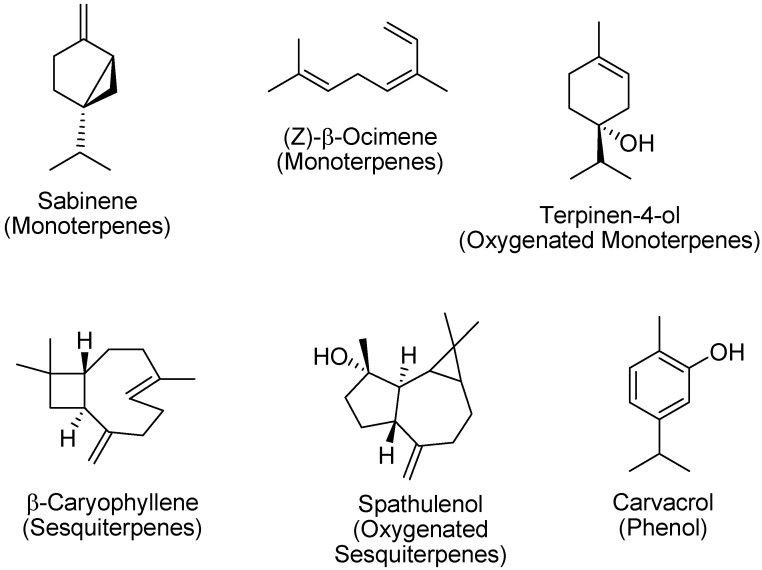
Chemical structures of the main constituents of *Origanum vulgare* essential oils.

The composition of essential oils obtained from plants subjected to two different treatments of cultivation and collected at full bloom is shown in Table 2, were they are listed in order of elution on the HP 5MS column and with the components grouped by biogenetic classes of compounds. Overall, 120 compounds representing 90.4%–93.9% of the oil were identified by GC and GC/MS. Although the total content of monoterpene hydrocarbons is similar for the matrices both in the fresh and in the dry state, there are differences in the content of individual components. Indeed, the oil from plants grown on single rows is rich in sabinene, while that from plants grown in double rows is richer in ocimenes. As for the oxygenated monoterpenes, terpinen-4-ol is the most abundant component. The total content in sesquiterpene hydrocarbons is higher in oils obtained from fresh matrices, and among them the most abundant are β-caryophyllene, germacrene D and α-humulene. Among the oxygenated sesquiterpenes, spathulenol is the most abundant in all oils and its content is greater in the dried samples. As for the phenol content, carvacrol is the main constituent and its concentration increases in the dry matrix. The structures of the main constituents of the essential oils are reported in Figure 1.

### 2.3. Antimicrobial Activity

Table 3 reports the Minimum Inhibitory Concentration (MIC) and the Minimum Bacterial Concentration MBC values of the essential oils against ten Gram positive and Gram negative bacterial strains, selected among those known to cause respiratory, gastrointestinal, skin and urinary disorders in humans.

The essential oils showed mild activity mainly against the Gram-positive pathogens, among which *Bacillus cereus* and *B. subtilis* were the most affected. Among Gram-negative bacteria, only *E. coli* was affected by the oils 2 and 4. The higher activity of oils 2 and 4 compared to that of oils 1 and 3 could be ascribed to the higher content of carvacrol, in agreement with the reported relationship between activity and the presence of some phenolic components, especially thymol and carvacrol [28,29].

**Table 3:** MIC and MBC* values (µg/mL) of essential oils from *Origanum vulgare* L. subsp. *vulgare* L. at full bloom and MIC of reference antibiotic.

Bacterial strain	Oregano essential oil samples				G
	Single row		Binate row		
	Fresh (1)	Dried (2)	Fresh (3)	Dried (4)	
*Bacillus cereus* ATCC 11778	50 (100)	50	50 (100)	50	1.56
*Bacillus subtilis* ATCC 6633	50 (100)	50	50 (100)	50	1.56
*Staphylococcus aureus* ATCC 2592	50 (100)	50 (100)	100	50 (100)	3.12
*Staphylococcus epidermidis* ATCC 12228	50 (100)	50 (100)	100	50 (100)	6.25
*Streptococcus faecalis * ATTC 29212	100	50 (100)	100	50 (100)	>100
*Escherichia coli* ATCC 25922	100	50 (100)	100	50 (100)	3.12
*Proteus mirabilis* ATCC 25933	100	100	>100	100	100
*Proteus vulgaris* ATCC 13315	>100	100	>100	100	100
*Pseudomonas aeruginosa* ATCC 27853	>100	>100	>100	>100	12.5
*Salmonella typhi Ty2* ATCC 19430	>100	>100	>100	>100	>100

* MBC values are reported in brackets when different from MIC values; G: Gentamycin.

## 3. Experimental

### 3.1. Field Trial

The research started at the beginning of 2007 on plants of *Origanum vulgare* L. subsp. *vulgare* L. (sin. *O. vulgare* L.) [26] coming from a commercial nursery after three years from the transplantation in the farm of CRAA “Improsta” in a plane area of Southern Italy (Eboli, Salerno) on a deep clay sandy soil having pH 8.3, low in nitrogen, (0.84‰), well provided in P_2_O_5_ (38.4 ppm) and rich in K_2_O (423 ppm).

The experimentation compared two plants distribution: single row (distance of 0.40 m between the rows and 0.50 m between the plants on the row corresponding to 5 plants m^−2^) and binate row (distance of 1.20 m between the bine and 0.20 m in the bine, 0.50 m between the plants on the row corresponding to 2.9 plants m^−2^). The distance between the bine was chosen to evaluate the possibility to introduce the mechanical control of weeds. For each scheme four rows of 15 m long were considered as replications. The area was equipped with a drop irrigation system which was used to facilitate the transplanting of the oregano plants and to ensure their normal growth in times of drought. Every year the plants were fertilized with 40 units of nitrogen per hectare during the third ten days of March.

The plant growth was analyzed in correspondence of significant phases of the biological cycle by measuring the height of the vegetation and the width of the row until the ground covering with 10 measurements per plot. Moreover, fresh epigeous biomass was determined by collecting samples from a section of 50 cm of the single and binate rows for every replication.

The incoming Photosynthetically Active Radiation (PAR) was measured using a line quantum sensor from Decagon Device Inc. (Pullman, WA, USA) placed perpendicularly to the rows above the canopy (PARi) and at the soil level (PARt); moreover it was placed also face downwards to measure reflectance of the canopy (PARr) and of the soil (PARs). Ten data were collected for every parameter in the central hours of the day and they have been considered representative of the whole day [30]. The data were used to calculate PAR absorbed from the crop [31].

The essential oil content was determined by sampling three representative samples of the fresh epigeous biomass for every determination Data of growth were evaluated by ANOVA and the mean values were separated by LSD at *p* ≥ 5%.

### 3.2. Essential Oil Extraction and Analysis

Aliquots (25 g) of samples collected at full bloom both for single and binate rows were finely shredded and then submitted to hydrodistillation for 3 h using *n*-hexane as solvent and a Clevenger apparatus according to the method recommended in the *European Pharmacopoeia* [32]. The oils were dehydrated by anhydrous sodium sulfate and kept in a cool (+4 °C) and dark place prior to analysis. In addition, the same samples air dried at room temperature were submitted to hydrodistillation and analyzed.

The oils were analyzed at the Department of Pharmacy of the University of Naples “Federico II. The GC analysis was carried out with a Perkin-Elmer (Perkin-Elmer, Shelton, CT, USA) Sigma 115 gas chromatograph) equipped with a flame ionization detector (FID) and two stationary phases of different polarity: HP-5 MS (30 m length × 0.25 mm internal diameter × 0.25 μm film thickness) or HP Innowax polyethylene glycol capillary column (50 m length × 0.20 mm internal diameter × 0.33 μm film coating) fused silica columns, respectively. Helium was the carrier gas at 1.0 mL min^−1^. Oven temperature was programmed as follows: initial oven temperature was set at 40 °C (held for 5 min), raised to 250 °C at 2 °C min^−1^ and held for 5 min and finally increased at 275 °C at 5 °C min^−1^. Injector and detector temperatures were set at 250 °C and 280 °C, respectively. Diluted samples (1/100 in *n*-pentane, v/v) of 1*.*0 μL were injected in the split mode (split ratio 1:100). The analysis was repeated twice for each sample. For GC/MS analysis was used an Agilent 6850 Ser. II apparatus, fitted with a fused silica HP-5MS capillary column (30 m × 0.25 mm i.d.; 0.25 μm film thickness), coupled to an Agilent Mass Selective Detector MSD 5973; ionization voltage 70 eV; electron multiplier energy 2,000 V. Mass units were monitored from 35 to 450 amu. The same column temperature programme was applied as in GC analysis; injector and detector MS transfer line temperatures were set at 250 °C and 280 °C, respectively. Identification of constituents was made by comparing their relative retention index (K_i_) determined with reference to homologous series of *n*-alkanes (C_8_-C_30_) under identical experimental condition in both polar and non-polar columns, with either those of the literature [33,34], co-injection with standards and by matching recorded mass spectra with data published in the literatures [33,35,36] and by comparing them with reference spectra in the computer libraries (NIST 02 and Wiley 275). Quantification was computed by electronic integration of the FID peak areas without corrections for FID response.

### 3.3. Antimicrobial Assay

The antibacterial activity was evaluated by determining the minimum inhibitory concentration (MIC) and the minimum bactericidal concentration (MBC) using the broth dilution method [37]. Ten bacteria species, selected as representative of the class of Gram positive and Gram negative, were tested: *Bacillus cereus* (ATCC 11778), *Bacillus subtilis* (ATCC 6633), *Staphylococcus aureus* (ATCC 25923), *Staphylococcus epidermidis* (ATCC 12228), *Streptococcus faecalis* (ATTC 29212), *Escherichia coli* (ATCC 25922), *Proteus mirabilis* (ATCC 25933), *Proteus vulgaris* (ATCC 13315), *Pseudomonas aeruginosa* (ATCC 27853) and *Salmonella typhi Ty2* (ATCC 19430). The strains were maintained on Tryptone Soya agar; for the antimicrobial tests, Tryptone Soya broth was used. In order to facilitate the dispersion of the oils in the aqueous nutrient medium they were diluted with Tween 20 at a ratio of 10%. Each strain was tested with samples that were serially diluted in broth to obtain concentrations ranging from 100 μg/mL to 0.8 μg/mL. The samples were previously sterilized with Millipore filter of 0.45 mm. The samples were stirred, inoculated with 50 μg/mL of physiologic solution containing 5 × 10^6^ microbial cells, and incubated for 24 h at 37 °C. The MIC value was determined as the lowest concentration of the sample that didn’t permit any visible growth of the tested microorganism after incubation. Control containing only Tween 20 instead of the essential oil was not toxic to the microorganisms. As positive controls were used cultures containing only sterile physiologic solution Tris buffer. MBC was determined by subculture of the tubes with inhibition in 5 mL of sterile nutrient broth. After incubation at 37 °C the tubes were observed. When the germs did not grow, the sample denoted a bactericidal action. Oil samples were tested in triplicate. Gentamycin was used as standard antibacterial agent. Sterile distillated water and medium served as a positive growth control.

## 4. Conclusions

The results reported in this manuscript highlight the influence of different plant distributions on the growth of oregano. In the binate rows treatment, the vegetation didn't completely cover the soil even at the stage of maximum growth due to the high distance between the bine (120 cm) and, consequently, the values of the fresh biomass per plant were higher. On the contrary, the values of fresh biomass/m^2^ were always higher in the single row treatment because the greatest fresh biomass per plant in the binate row treatment was not sufficient to make up for the lower number of plants/m^2^. The essential oil percentages on fresh biomass were higher for the binate row treatment only at the full bloom and this can be explained by the high ability of the vegetation to intercept the PAR in both treatments. Moreover, the conditions of growth leading to a greater biomass/plant in the binate treatment contributed also to increase the essential oil percentage.

The essential oil content of fresh biomass is influenced by the conditions of plant growth and it increases during the biological cycle, reaching the maximum values at the full bloom. The oils extracted from matrices, collected in this stage and air dried, contained a fairly good amount of phenols and other components, exerting antibacterial activity. This finding confirms the interest of these plants for pharmaceutical and nutraceutical applications and the results of the present study could provide useful information to better exploit oregano plants for these purposes.

## References

[1] De Feo V., de Falco E., Nicolella E., Roscigno G. (2005). First observation on a collection of aromatic plants in a plain of the Campania region (Southern Italy). Acta Hort..

[2] Marzi V., Tedone L. (2006). Fattori climatici e socio economici nell’evoluzione del paesaggio agrario e forestale in ambiente mediterraneo. Ital. J. Agron..

[3] Gonzáles-Tejero M.R., Casares-Porcel M., Sánchez-Rojas C.P., Ramiro-Gutiérrez J.M., Molero-Mesa J., Pieroni A., Giusti M.E., Censorii E., de Pasquale C., Della A. (2008). Medicinal plants in the Mediterranean area: Synthesis of the results of the project RUBIA. J. Ethnopharmacol..

[4] Hadjichambis A.C., Paraskeva-Hadjichambi D., Della A., Giusti M.E., de Pasquale C., Lenzarini C., Censorii E., Gonzáles-Tejero M.E., Sánchez-Rojas C.P., Ramiro-Gutierrez J.M. (2008). Wild and semi-domesticated food plant consumption in seven circum-Mediterranean areas. Int. J. Food Sci. Nutr..

[5] Senatore F. (2000). Oli Essenziali: Provenienza, Estrazione ed Analisi Chimica.

[6] Sahin F., Gulluce M., Daferera D., Sokmen A., Polissiou M., Agar G. (2004). Biological activities of the essential oils and methanol extract of *Origanum vulgare* subsp. *vulgare* in the Eastern Anatolia Region of Turkey. Food Control.

[7] Faleiro L., Miguel G., Gomes S., Costa L., Venâncio F., Teixeira A., Figueiredo C., Barroso J.G., Pedro L.G. (2005). Antibacterial and antioxidant activities of essential oils isolated from *Thymbra capitata* L. (Cav.) and *Origanum vulgare*L.. J. Agric. Food Chem..

[8] Souza E.L., Stamford T.L.M., Lima E.O., Trajano V.N. (2007). Effectiveness of *Origanum vulgare* L. essential oil to inhibit the growth of food spoiling yeasts. Food Control.

[9] Saraç N., Uğur A., Duru M.E., Varol Ö. (2009). Antimicrobial activity, antioxidant activity and chemical composition of *Origanum onites* L. and *Origanum vulgare* L. subsp. *hirtum* (Link) Ietswaart from Mugla (Turkey). Acta Hortic..

[10] Coelho da Costa A., Cavalcanti dos Santos B.E., Santos F.L., Lima E.O. (2009). Antibacterial activity of the essential oil of *Origanum vulgare* L. (Lamiaceae) against bacterial multiresistant strains isolated from nosocomial patients. Rev. Bras. Farmacogn..

[11] Tommasi L., Negro C., Miceli A., Mazzotta F. (2009). Antimicrobial activity of essential oils from aromatic plants grown in the Mediterranean area. J. Essent. Oil Res..

[12] Dadalioglu I., Evrendilek G.A. (2004). Chemical composition and antibacterial effects of essential oils of Turkish oregano (*Origanum minutiflorum*), bay laurel (*Laurus nobilis*), Spanish lavender (*Lavandula stoechas* L.), and fennel (*Foeniculum vulgare*) on common foodborne pathogens. J. Agric. Food Chem..

[13] Baydar H., Sagdic O., Özkan G., Karadogan T. (2004). Antibacterial activity and composition of essential oils from *Origanum*, *Thymbra* and *Satureja* species with commercial importance in Turkey. Food Control.

[14] Vardar-Ünlü G., Ünlü M., Dönmez E., Vural N.  (2007). Chemical composition and *in vitro* antimicrobial activity of the essential oil of *Origanum minutiflorum* O Schwarz & PH Davis. J. Sci. Food Agric..

[15] Bouhdid S., Skali S.N., Idaomar M., Zhiri A., Baudoux D., Amensour M., Abrini J. (2008). Antibacterial and antioxidant activities of *Origanum compactum* essential oil. Afr. J. Biotechnol..

[16] Başer K. (2008). Biological and pharmacological activities of carvacrol and carvacrol bearing essential oils. Curr. Pharm. Design.

[17] Kokkini S., Padulosi S. (1997). Taxonomy, diversity and distribution of *Origanum* species. Proceedings of IPGRI International Workshop on Oregano.

[18] Giuliani C., Maggi F., Papa F., Maleci Bini L. (2013). Congruence of phytochemical and morphological profiles along an altitudinal gradient in *Origanum vulgare* subsp. *vulgare* from Venetian Region (NE Italy). Chem. Biodivers..

[19] Berghold H., Wagner S., Mandi M., Thaller A., Muller M., Rakowitz M., Pasteiner S., Boechzelt H. (2008). Yield, content and composition of the essential oil of 5 oregano strains (*Origanum vulgare* L.) depending on the developmental stage. Z. Arznei Gewuerzpfla.

[20] Azizi A., Yan F., Honermeier B. (2009). Herbage yield, essential oil content and composition of three oregano (*Origanum vulgare* L.) populations as affected by soil moisture regimes and nitrogen supply. Ind. Crop. Prod..

[21] Bernestein N., Chaimovitch D., Dudai N. (2009). Effect of irrigation with secondary treated effluent on essential oil, antioxidant activity, and phenolic compounds in oregano and rosemary. Agronomy J..

[22] Dordas C. (2009). Foliar application of calcium and magnesium improves growth, yield, and essential oil yield of oregano (*Origanum vulgare* subsp. *hirtum*). Ind. Crop. Prod..

[23] Halva S., Craker L.E., Simon J.E., Charles D. (1992). Light quality, growth and essential oil in dill (*Anethum graveolens* L.). J. Herbs Spices Med. Plants.

[24] Piccaglia R. (1998). Aromatic plants: A world of flavouring compost. Agro-Food Ind. Hi-Tech.

[25] Leto C., Carrubba A., Trapani P. Effetti della densità d’impianto sulla coltivazione dell’origano (*Origanum heracleoticum* L.) in due ambienti siciliani. Proceedings of Coltivazione e miglioramento di piante officinali.

[26] Ietswaart J.H. (1980). A Taxonomic Revision of the Genus Origanum (Labiatae).

[27] Marzi V., Padulosi S. (1997). Agricultural practices or oregano. Proceedings of IPGRI International Workshop on Oregano.

[28] Lambert R.J.W., Skandamis P.N., Coote P., Nychas G.J.E. (2001). Study of the minimum inhibitory concentration and mode of action of oregano essential oil, thymol, and carvacrol. J. Appl. Microbiol..

[29] Rhayour K., Bouchikhi T., Tantaoui-Elaraki A., Sendide K., Remmal A. (2003). The mechanism of bactericidal action of oregano and clove essential oils and of their phenolic major components. J. Essent. Oil Res..

[30] Guiducci M., Antognoni A., Benincasa P. (1993). Movimento del fogliame, intercettazione ed utilizzazione della luce in colture diverse in funzione della disponibilità idrica. Riv. Agron..

[31] Ercoli E., Mensuali A., Malorgio F., Serra G. (1992). Interception of photosynthetically active radiation, growth and production of bush bean (*Phaseolus vulgaris* L.). Agric. Mediterr..

[32] EDQM (2005). European Pharmacopoeia.

[33] Jennings W., Shibamoto T. (1980). Qualitative Analysis of Flavour and Fragrance Volatiles by Glass Capillary Gas Chromatography.

[34] Davies N.W. (1990). Gas chromatographic retention indices of monoterpenes and sesquiterpenes on methyl silicone and Carbowax 20M phases. J. Chromatogr..

[35] Adams R.P. (2007). Identification of Essential Oil Components by Gas Chromatography/Quadrupole Mass Spectroscopy.

[36] The Mass Spectrometry Data Centre (1983). Eight Peak Index of Mass Spectra.

[37] Barry A. (1976). The Antimicrobial Susceptibility Test: Principles and Practices.

